# Ophthalmic Manifestations in Fabry Disease: Updated Review

**DOI:** 10.3390/jpm13060904

**Published:** 2023-05-27

**Authors:** Gloria Gambini, Luca Scartozzi, Federico Giannuzzi, Matteo Mario Carlà, Francesco Boselli, Tomaso Caporossi, Umberto De Vico, Antonio Baldascino, Stanislao Rizzo

**Affiliations:** 1 Ophthalmology Department, Fondazione Policlinico Universitario A. Gemelli, IRCCS, 00168 Rome, Italy; gambini.gloria@gmail.com (G.G.); luca.scartozzi01@icatt.it (L.S.); federico.giannuzzi@gmail.com (F.G.); francescoboselli@outlook.it (F.B.); umbertodevico@gmail.com (U.D.V.); antonio.baldascino@policlinicogemelli.it (A.B.); stanislao.rizzo@policlinicogemelli.it (S.R.); 2 Ophthalmology Department, Catholic University “Sacro Cuore”, 20123 Rome, Italy; tomaso.caporossi@gmail.com; 3 Vitreoretinal Surgery Unit, Fatebenefratelli Isola Tiberina-Gemelli Isola Hospital, 00186 Rome, Italy

**Keywords:** Fabry disease, cornea verticillata, vortex keratopathy, vascular tortuosity, hyper-reflective foci, foveal avascular zone, focal electroretinography

## Abstract

Fabry disease (FD) is an X-linked lysosomal storage disorder, causing Gb-3 (globotriaosylceramide) buildup in cellular lysosomes throughout the body, in particular in blood vessel walls, neuronal cells, and smooth muscle. The gradual accumulation of this glycosphingolipid in numerous eye tissues causes conjunctival vascular abnormalities, corneal epithelial opacities (cornea verticillata), lens opacities, and retinal vascular abnormalities. Although a severe vision impairment is rare, these abnormalities are diagnostic indicators and prognostics for severity. Cornea verticillata is the most common ophthalmic feature in both hemizygous men and heterozygous females. Vessel tortuosity has been linked to a faster disease progression and may be useful in predicting systemic involvement. New technologies such as optical coherence tomography angiography (OCTA) are useful for monitoring retinal microvasculature alterations in FD patients. Along with OCTA, corneal topographic analysis, confocal microscopy, and electro-functional examinations, contributed to the recognition of ocular abnormalities and have been correlated with systemic involvement. We offer an update regarding FD ocular manifestations, focusing on findings derived from the most recent imaging modalities, to optimize the management of this pathology.

## 1. Introduction

Fabry disease (FD) is a rare X-linked lipid storage disorder characterized by a deficiency or absence of the lysosomal enzyme α galactosidase-A (α gal-A), causing progressive accumulation of globo-triaosylceramide (Gb-3) in cells throughout the body. Before the availability of hemodialysis and enzyme replacement therapy (ERT), premature death before 50 years of age often resulted from stroke, heart attack, or renal failure [1].

Ophthalmological manifestations are common in Fabry disease and usually do not cause significant visual impairment or other ocular symptoms. Some manifestations may provide information on the progression of the disease. Some studies suggest a correlation between the severity of the disease and the presence of ocular signs, such as cornea verticillate, vessel tortuosity, and cataracts [2,3].

Our review focuses on recent findings from several imaging modalities, such as optical coherence tomography angiography (OCTA), which can highlight correlations and predictivity with other systemic manifestations.

## 2. Epidemiology and Genetics

FD is a pan-ethnic disease with a wide spectrum of heterogeneous clinical phenotypes; multiple mutations have been documented. In newborn screening studies, a prevalence of 1:1250 was reported [4]. In Italy, the frequency is reported at approximately 1 in 3100 newborns [5], while in Taiwan, the frequency in newborn males may be as high as 1 in 1500 [6]. In the general population, the incidence ranges from 1 in 40,000 to 1 in 117,000 [7,8,9].

FD is caused by reduced or absent levels of α gal-A in lysosomes, due to mutations in the gene GLA. This gene, mapped to the region q22.1 of the X chromosome, encodes a homodimeric glycoprotein that hydrolyzes the terminal alpha-galactosyl moieties from glycolipids and glycoproteins. The severity of the disease is proportional to the amount of α-gal-A activity [10,11], with over 585 documented pathogenic mutations in the GLA gene causing non-functionality of the enzyme [12,13].

Three distinct phenotypes of FD are recognized:

- Males with no α-gal-A activity. More than 40 mutations of α-gal-A (no α-gal-A activity) have been correlated with the classic phenotype [11,14,15,16]. Individuals with this phenotype develop the full spectrum of clinical signs and symptomatology, with symptom onset in childhood. This mutation occurs in approximately 1 in approximately 37,000 to 60,000 males [15].
- Males with non-classic or atypical FD mutations, resulting in different manifestations. Some gene mutations result in partial α-gal-A activity. These patients may experience symptoms in childhood, but they do not manifest the full spectrum of FD. Compared to the classic phenotype, these variants seem to have more cases of midlife cardiac and renal disease [11].
- Female carriers exhibit a variety of manifestations. Although they were once considered carriers only, females are now recognized as being affected with variable penetrance [11]. The phenotypic variation seen in heterozygotes is caused by random X-inactivation, or lyonization, allowing traits of the mutated X chromosome to be expressed to varying degrees [17,18,19,20].

Males are hemizygotes because they inherit one mutated X-chromosome from their mothers. Females are known as heterozygotes because they inherit the mutated X-chromosome from either parent.

## 3. Pathophysiology

Fabry disease causes Gb-3 accumulation in many cell types, with lysosomal storage of Gb-3 beginning in utero and increasing over time [21,22,23]. Nonetheless, as little as 5–10% of residual enzyme activity is sufficient to prevent clinically significant accumulation of Gb-3 [24].

The progressive storage of these molecules leads to cellular dysfunction and secondary inflammation or fibrosis. The vascular endothelium is one of the main tissues involved in the disease, causing poor perfusion and tissue damage in organs with high vascularity, such as kidneys, heart, nervous system, eye structures, and skin alone or in combination [24,25].

## 4. Conjunctival Manifestations

The most characteristic ocular manifestations in FD are increased vessel tortuosity, venous vascular aneurysmal dilation, and ‘sludging’ of the blood in the small blood vessels of the conjunctiva. These changes can be seen in any conjunctival area, but they are most commonly located in the inferior bulbar region. Vessel tortuosity is more common in males than in females and has a significant correlation with the disease severity score (greater impairment of renal and cardiac function); Sodi et al. showed a tortuosity in 22% cases in females and 49% in males [2,26].

Several studies have highlighted that the histopathological abnormalities are caused by abnormal storage of Gb-3 in the endothelial cells, pericytes, and smooth muscle cells of the conjunctival vessel walls, leading to degenerative changes responsible for the weak mechanical resistance of the vessel walls to blood pressure and causing, over time, the anomalies in the blood vessel architecture. Similar deposits have been reported in all layers of the conjunctival epithelial cells, including the goblet cells [27,28,29,30].

Conjunctival varicosities appear between the second and third decades of life and are observed in nearly 100% of hemizygous males and about 50% of heterozygous females, in addition to those reported in the retina of those individuals [2,12,31]. The parallelism between conjunctival and retinal alterations suggests abnormal blood flow in vessels throughout the body and, therefore, highlights the usefulness of predictors of more severe systemic involvement such as loss of sympathetic tone (a dysautonomic manifestation of FD) or occlusion vasculopathy [26,32,33,34,35].

Importantly, Mastropasqua and colleagues, and Falke and coworkers, found that conjunctival lymphangiectasia (CL) represents a common but under-recognized ocular manifestation of FD, observed in almost 80% of cases despite long-term enzyme replacement therapy [36,37]. Clinical presentations included single cysts, beaded dilatations, and areas of conjunctival oedema, with lesions located within 6 mm of the corneal limbus. Those abnormalities are often accompanied by peripheral lymphoedema, dry eye syndrome, and conjunctival chemosis [38,39].

In vivo confocal microscopy (IVCM) studies showed an irregular epithelial morphology, with poorly distinguishable cell borders and the presence of reflective non-homogeneous material, widely distributed or interspersed between areas of normal cellular architecture among less reflective epithelium [40,41]. The tarsal conjunctival epithelium was visualized in all patients and was the most evident pathologic finding. The presence of roundish hyper-reflective intracellular structures involving most of the cells was the main observed feature [36,37]. In studies by Mastropasqua and Falke, the tarsal conjunctiva showed two different types of round hyper-reflective intracellular structures in most cells. The two types were papillary and columnar distribution, following the conjunctival epithelial folding, and were the same in both males and females [36,37].

## 5. Corneal Manifestations

Vortex keratopathy (VK), also known as cornea verticillata, the most typical ocular sign in Fabry disease, was first described by Fleischer and colleagues in 1910 [42], while in 1925, Weicksel and colleagues recognized VK as being related to FD [43]. In 1968, while studying a family with FD and various cases of VK, Franceschetti described an X-linked recessive model of inheritance [44].

**Figure 1:**
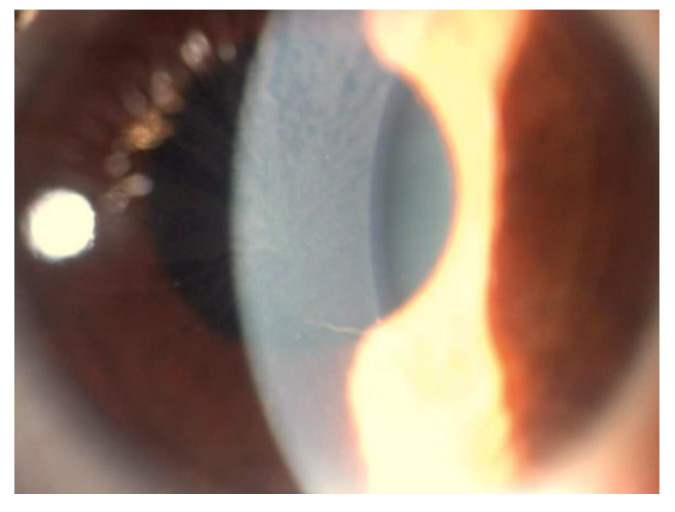
Picture of a typical form of cornea verticillate in a patient affected.

VK consists of bilateral whorl-like opacities with a vortex pattern located in the superficial corneal layers, most commonly in the inferior corneal area. These opacities are typically cream-colored, ranging from whitish to golden-brown. In the early stages, the opacities may form fine horizontal lines, but they later develop into curving lines, radiating from a point below the center of the cornea, forming small whorls, before becoming almost straight at the periphery. The pattern and location of these corneal deposits may be related to the influence of ocular hydrodynamics, periodic blinking, ocular magnetic fields, and/or the centripetal movement of the renewing epithelial cells from the periphery toward the center of the cornea [45,46,47] (Figure 1).

VK is evident in most hemizygous males (73%) and heterozygous females (77%) [2]. Hence, they are usually considered to be the most reliable ophthalmological marker of Fabry disease. Occasional patients with a genetic diagnosis of Fabry disease do not exhibit the corneal changes. Moiseev and colleagues found that VK was not associated with the severity of the disease [48]. Furthermore, Pitz and coworkers studied VK and specific genetic mutations; they found null mutation (male, 77%; female, 65%), missense mutation (male, 79%; female, 67%), mild missense mutation (male, 17%; female, 23%) and the p.N215S mutation (male, 15%; female, 16%) [3].

Some reports of a sub-epithelial corneal haze have been described, in addition to the more typical whorl-like opacities [49]. In most of these patients, this brownish haze, or more rarely grey or whitish haze, is diffuse and involves the entire cornea, but in some individuals, it is limited to the central or limbal corneal area. Rahman and Orssaud postulated that the haze is an early manifestation of FD, and/or a natural evolution of the vortex opacities [49,50].

### 5.1. Histopatological Findings

Histopathological studies showed the presence of intra-epithelial deposits, consisting of dense laminated cytoplasmic inclusions, both membrane-bound and lying freely in the cytoplasm, in both hemizygous and heterozygous FD patients [51,52,53,54,55]. In a study of the cornea of a woman with FD, Weingeist and Blodi suggested that the diffuse accumulation of sphingolipids in the corneal epithelium might be responsible for the diffuse corneal haze, and the whorl-like pattern might be determined by a series of sub-epithelial ridges [56]. Recent ultrastructural investigations revealed a disruption of the normal pattern of the basement membrane without re-duplication of the basal lamina [55]. A possible corneal endothelium involvement in FD was suggested by the finding of pigment and corneal guttae on the endothelium in sporadic cases, but this has not been confirmed by other investigators [57].

### 5.2. Corneal Biomechanics

Biomechanical studies have provided evidence that the accumulation of sphingolipids in the cornea affects static and dynamic responses, in eyes with VK. Cankurtaran et al., using corneal tomography, demonstrated that FD patients have statistically significant higher corneal densitometry values in all corneal concentric zones and layers, except the posterior 0–2 mm and posterior 2–6 mm zones, as compared to healthy eyes; thus, VK is associated with increased light backscattering and reduced corneal transparency [58]. Moreover, corneal biomechanical analysis conducted with Corvis ST (Oculus Optikgerate Gmbh, Wetzlar, Germany) showed increased corneal stiffness in VK, probably due to the stromal accumulation of sphingolipids [58]. Koh et al., through the use of quantitative contrast sensitivity evaluations, found clear functional deficits in contrast sensitivity measurements, despite normal visual acuities, in patients with VK [59].

### 5.3. In Vivo Confocal Microscopy

IVCM was studied in FD patients by Leonardi and colleagues, who found epithelial deposits in 89% of FD patients’ eyes, compared to 32% visualization with slit-lamp examination [60]. Moreover, a substantial decrease in the length, quantity, and density of corneal subepithelial nerve fibers, as well as an elevated grade of tortuosity, was reported [60,61,62], confirming the existence of an FD-related corneal neuropathy in addition to systemic small fiber neuropathy [63]. When compared to healthy subjects, an assessment of ocular symptoms in 75 patients with FD indicated a statistically significant higher incidence of dryness, blurry/dim vision, and halos around lights [64]. A decrease in corneal sensitivity was found using a contact corneal esthesiometer (Cochet-Bonnet; Luneau, France), validating the suspicion of corneal nerve involvement in FD and a probable link to tear film malfunction [65].

### 5.4. Stromal and Endothelial Disfunction

Corneal endothelial cell abnormalities have been documented by Bitirgen et al. and correlated with disease severity as measured with the Mainz Severity Score Index (MSSI). In addition, this study demonstrated an increased density of dendritic cells in the central cornea [65]. Moreover, a flow cytometry analysis in FD patients showed a reduction in circulating dendritic cells in peripheral blood samples, suggestive of increased extravasation and migration to peripheral tissues, such as the central cornea. This mechanism, described for FD, is common in various neuropathies and during inflammation [66].

### 5.5. Differential Diagnosis of Vortex Keratopathy

VK is often associated with multiple myeloma [67], monoclonal gammopathy of undetermined significance (MGUS) [41], and long-term therapy with any of the following drugs: amiodarone [68], chloroquine [69], subconjunctival gentamicin, gold, non-steroidal anti-inflammatory drugs (NSAID) such as indomethacin, naproxen, ibuprofen, phenothiazines, tamoxifen, and monobenzone (topical skin ointment).

The IVCM revealed hyper-reflective intracellular inclusions in basal epithelial cells in both amiodarone-induced and FD’s VK. While these two conditions cannot be distinguished with conventional slit-lamp microscopy, confocal laser-scanning microscopy allowed the differentiation between the two etiologies and revealed corneal changes before slit-lamp microscopy in several studies [37,40,70].

In amiodarone-induced keratopathy, deposits were more reflective and of different sizes with increasing time on therapy [70]. The highly reflective epithelial cells were initially found at the center of the cornea and subsequently spread to the periphery. However, even after the keratopathy had progressed, the highly reflective epithelial cells were not visible at the limbus, implying that during their centripetal movement, ocular epithelial cells endocytosed amiodarone from the tear film [40]. This assessment was supported by the delayed observation of microdots in the stroma and endothelial cells, after being detected in epithelial cells, and may be due to the fact that penetration of the tear fluids through the stroma and endothelium required more time, compared to the epithelium; in vivo confocal microscopy was able to detect the endocytosed amiodarone as the highly reflective material into the corneal epithelial cells only after 1 to 3 months of therapy [68,71,72].

In FD, the highly reflective epithelial cells were consistently observed extending from the limbus to the central cornea. This suggested that Gb-3 is deposited in the lysosomes of limbal epithelial stem cells and that the limbal epithelial stem cells with Gb-3 deposits moved toward the cornea’s center [40].

Eventually, microdot changes in the anterior stroma were more prevalent in patients receiving amiodarone, but do not correlate with the simultaneous presence of cornea verticillate [37,70].

## 6. Lens Manifestations

**Figure 2:**
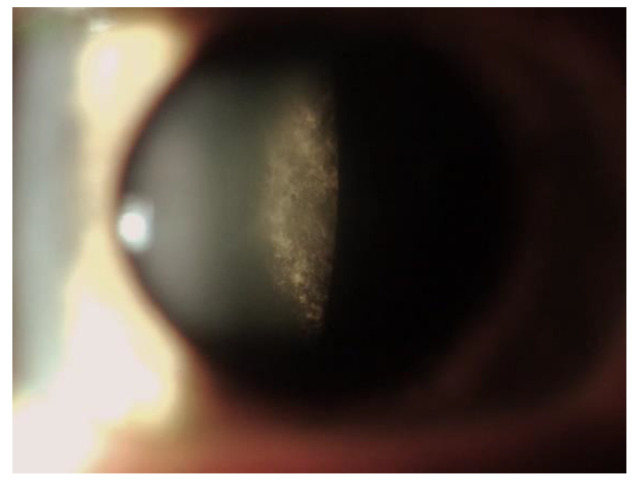
Picture of a typical form of cataract in a patient affected.

FD cataracts are found more frequently in males than in females and strongly correlate with disease severity, arising in the second decade of life (up to 70% of males) [2,31,50] (Figure 2). The two most common symptoms of lens opacities are radial posterior subcapsular cataract and anterior capsular or subcapsular cataract, which are often bilateral and wedge-shaped [73,74].

Lens opacification is caused by Gb-3 deposits in the lens epithelium and are best seen in retroillumination [27,31,75]. Two subtypes of cataracts have been identified, with different characteristics: (1) The posterior subcapsular cataracts represent the most frequent type and have been reported to be specific for the disease (also called “classic Fabry cataracts”), causing the most detrimental effects on visual function and often requiring surgical intervention. It consists of off-axis or dendritic whitish opacities along the posterior lens sutures near the posterior capsule, with a spoke-like appearance [31,75]. The next subtype—(2) the anterior capsular and subcapsular opacities, so-called ‘propeller’ cataracts—are generally bilateral and wedge-shaped, with a radial distribution from the equator to the center of the anterior capsule [76].

## 7. Retinal Manifestations

**Figure 3:**
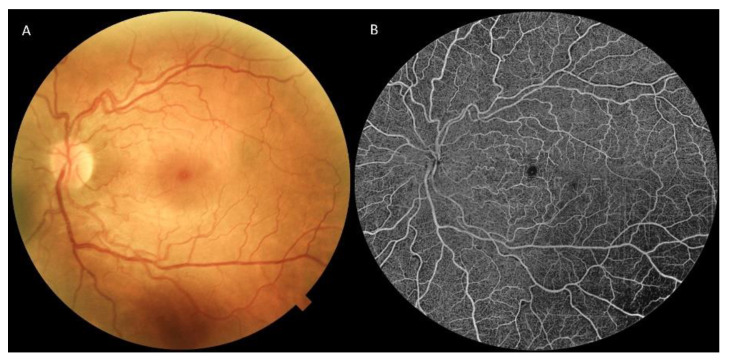
Posterior pole retinography (**A**) and OCTA (**B**) image of a Fabry disease patient, highlighting marked vascular tortuosity. OCTA image is segmented at the SVP, making FAZ clearly visible. OCTA = optical coherence tomography angiography, SVP = superficial vascular plexus, FAZ = foveal avascular zone.

FD leads to an increase in vascular tortuosity, in both arteries and veins. Ophthalmoscopy can detect retinal vascular tortuosity, but diagnostic tools are fundamental to define its stage and features (Figure 3).

### 7.1. Retinography

Sodi et al. evaluated the tortuosity using retinography (Retinographs TF 450 Plus, Carl Zeiss, Dublin, CA, USA) and using three parameters: sum of angles metric (SOAM), product of angle distance (PAD), and triangular index (I2e) [26]. They reported that retinal vascular tortuosity was higher in comparison with the control group and, moreover, retinal vessel tortuosity and conjunctival tortuosity were not correlated, suggesting the presence of morphological and physio-pathological distinctions between the two. For all outcome variables, no differences between males and females were reported [26]. Sher et al., in contrast with the findings previously reported, showed that hemizygous men had significantly greater and more severe retinal vascular alterations (70%), compared with 25% prevalence in the heterozygous females [31].

### 7.2. Fluorescein and Indocyanine Green Angiography

Ohkubo et al., using fluorescein angiography (FA), showed that the choroidal filling and intra-retinal circulation times were both delayed [77]. Vascular tortuosity was found in about 60% of male patients, in which the involvement was usually bilateral and occurred in both eyes in a symmetrical pattern [50]. Endothelial cells, smooth muscle, and pericytes are affected by the gradual deposition of non-catabolized glycosphingolipids, resulting in vessel wall thickening and lumen narrowing [78,79,80]. These vascular changes have been linked to optic neuropathy [81,82], retinal ischemia, and central retinal artery occlusion or thrombotic events [83].

Furthermore, Dantas et al., with the use of indocyanine green angiography (ICGA), demonstrated that FD patients suffered from a choroidal vasculature impairment, displaying a moth-eaten look with regions of hypoperfusion, gaps in vascular continuity, and vessel looping [84].

### 7.3. Optical Coherence Tomography and Optical Coherence Tomography Angiography

Structural changes in the macular areas of patients with FD have been analyzed with the ease of optical coherence tomography (OCT). Atiskova and colleagues found the presence of inner retinal hyper-reflective foci (HRF) using SD-OCT imaging (Spectralis OCT, Heidelberg Engineering, Heidelberg, Germany) of the macula. The HRF foci were primarily in the retinal nerve fiber layer and outer plexiform layer. They hypothesized that the deposition of endothelial glycosphingolipids could be a potential explanation for the HRF presence, resulting in a pathologically hyper-reflective capillary plexus of the inner retina. Nevertheless, the clinical implications of this finding are yet to be defined, since HRF grading seemed not to correlate with disease severity [85].

Recently, FD retinal features focused on optical coherence tomography angiography (OCTA) findings, an objective and non-invasive tool for the evaluation of the retinal microvascular changes [86].

Bacherini et al. investigated changes in retinal microvascularization in affected patients using an SD 3 × 3 mm OCTA (RS-3000 Advance 2 OCT; NIDEK Co. Ltd., Gamagori, Japan) and found that the FD group had significantly decreased vascular density in both the superficial capillary plexus (SCP) and the deep capillary plexus (DCP) [87]. Nevertheless, the vascular perfusion indexes and foveal avascular zone (FAZ) parameters showed no significant changes in neither area, perimeter, nor circularity [87].

Both Hufendiek and Cakmak found a reduction of vessel density in both the foveal SCP and DCP, even with different instruments: the first using a 3 × 3 mm OCTA (Spectralis OCT2, Heidelberg Engineering GmbH, Germany), the latter using a 6 × 6 mm OCTA (RTVue XR Avanti, Opto-Vue, Inc., Fremont, CA, USA) [88,89]. In addition, Cakmak and coworkers also reported an enlargement of the FAZ, while showing no differences in the density of radial peripapillary capillaries when compared to healthy subjects [89].

Finocchio et al. evaluated 13 FD patients using a Spectral Domain 3 × 3 mm OCTA (RTVue XR Avanti, Optovue, Inc., Freemont, CA, USA), showing a reduction in DCP vascular density and a reduction in the FAZ area of both the SCP and DCP, when compared to healthy controls, but no differences in SCP vascular density [90]. Conversely, Cennamo et al. evaluated 54 patients with FD and 70 controls, showing a reduction in vessel density in the SCP but an increase in the DCP, using a 6 × 6 mm OCTA (RTVue XR Avanti, Optovue, Inc., Freemont, CA, USA) [91].

On the other hand, Minnella et al. demonstrated an increase in SCP vascular density but not in the other layers, and an enlargement of the FAZ area in both the SCP and DCP, using a swept source 4.5 × 4.5 mm OCTA (Topcon, Tokyo, Japan) [92].

A summary of the reported outcomes in recent years regarding OCTA analysis in FD patients is available in Table 1, showing contradictory results. The reasons for this disparity may depend on the fact that a major part of those studies had small sample sizes and frequently related to various degrees of disease, making these data difficult to compare. In addition, different OCTA devices with different scan widths were used, making the findings less comparable and bringing out the need for further studies with larger cohorts and standardized imaging protocols. In fact, while on one side the presence of microvascular impairment seems to be obvious in FD patients, both the correlation of this manifestation with systemic grading and the possible use of OCTA findings as predictors for disease evolution have still to be determined.

### 7.4. Adaptive Optics

In 2020, Sodi and colleagues used adaptive optics (rtx1; Imagine Eyes, Orsay, France) to study retinal vessels in individuals with FD, providing a non-invasive and objective method of assessing microvascular involvement [94]. They found that 78% of affected patients had para-vascular punctuate or linear opacities, most likely corresponding to accumulated sphingolipids, that were limited to the wall of precapillary arterioles in the least-affected individuals (discrete 5 to 10 µm spots), while there was widespread opacification of the wall of capillaries and first-order arteries in the more seriously affected patients. These deposits sometimes had a striated pattern, suggesting localization with vascular smooth muscle cells. Sodi et al. hypothesized that sphingolipids accumulation in mural cells may determine functional changes of the vascular wall, hence making it less resistant to blood pressure and consequently leading to increased tortuosity [94].

### 7.5. Electrofunctional Findings

The focal electroretinography (fERG) method was used to investigate the macular function, focusing on the outer and middle retinal functional activity. Minnella and coworkers found that the fERG amplitude was significantly reduced when compared to the control group (0.87 ± 0.41 vs. 2.22 ± 0.24 µV; t = −10.647, *p* < 0.001) [92]. This reduction, combined with the reported intact fERG phase values, suggests a preclinical dysfunction of the outer retinal layer that the OCT parameters when a concomitant reduction in the outer nuclear layer thickness occurs, yielding fERG as a useful method for detecting FD’s subclinical stages [92].

### 7.6. Correlation with Systemic Manifestations

In order to assess the clinical implications of retinal findings, Sodi and colleagues analyzed the correlation between retinal vascular tortuosity and the systemic stage of the disease, showing the absence of correlation [26]. Their results were in accordance with both Minnella et al. and Bacherini and coworkers. Minnella et al., evaluated the MSSI score, and the images obtained using DRI and Triton Swept-Source OCTA device (Topcon, Tokyo, Japan) [92]. Likewise, Bacherini et al. evaluated indicators of cardiac and renal impairment attributable to FD (maximum left ventricular wall thickness and glomerular filtration rate), compared with retinal tortuosity indexes, but did not find significant correlations between the OCTA and systemic parameters [87].

## 8. Other Ocular Findings

Pupillary abnormalities were found in FD patients. A study conducted by Bitirgen and coworkers evaluated light response alterations in FD patients, and correlated them with the severity of systemic autonomic symptoms (calculated with the Composite Autonomic Symptom Scale 31, COMPASS 31) [95]. The results highlighted significant reductions in the amplitude and duration of pupil contraction, and the latency of pupil dilation in patients with FD compared to control subjects. Moreover, the pupillomotor-weighted sub-score of the COMPASS 31 inversely correlated with the duration of pupil contraction and latency of pupil dilation, and directly correlated with the duration of pupil dilation, confirming the existence of an association between pupillary response abnormalities and the severity of autonomic symptoms.

FD patients have been reported to be affected, in a lower percentage of cases, from other craniofacial abnormalities, more commonly evidenced in males: periorbital fullness [96], prominent supraorbital ridges [96], bushy eyebrows [96], bilateral ptosis [96], broad nasal base [96], full lips [96], a prominent chin [96], prominent earlobes [96], and posteriorly rotated ears [97].

Less reported ocular manifestations are chronic uveitis [98], subfoveal choroidal neovascularization [93,99], disc edema, and optic atrophy caused by ischemic optic neuropathy with enlargement of the blind spot at the visual field [81].

## 9. Conclusions

FD is rare and manifests in a variety of ways in the early stages; the definite diagnosis is frequently delayed until the later stages. Fortunately, this condition has become more well known, and significant progress has been made in both the diagnostic and therapy options for this genetic condition.

Thanks to technological advancements in recent years, a more in-depth study of the ocular clinical consequences of this condition has been possible, providing measurable and reproducible parameters and offering new possibilities for preclinical recognition of ocular abnormalities.

Multicenter studies would be required in order to assess a standardized methodology in the evaluation of FD patients and to fully exploit newly introduced imaging tools, such as OCTA, for a comprehensive evaluation and management of this condition. Ocular specialists might significantly shorten diagnostic delays if they were more knowledgeable about Fabry disease, which would reduce the illness’s morbidity and death.

## Figures and Tables

**Table 1:** Review of recent reports regarding FD outcomes using OCTA.

Study Authors	OCTA Devices	Scan Width (mm)	No. of FD/Controls	Findings in FD Patients
Finocchio et al. (2018) [90]	Spectral Domain	3 × 3	13/13	↓ vascular density in DCP, = SCP; ↓ FAZ of SCP and DCP
Hufendiek et al. (2018) [88]	Spectral Domain	3 × 3	10/10	↓ flow density in DCP, SCP and choriocapillaris
Baur et al. (2018) [86]	Swept Source	3 × 3	14/8	↓ vascular density in DCP, = in SCP
Cennamo et al. (2019) [91]	Spectral Domain	6 × 6	54/70	↓ vascular density in SCP, ↑ in DCP
Minnella et al. (2019) [92]	Swept Source	4.5 × 4.5	20/17	↑ vascular density in SCP, = in DCP ↑ FAZ in SCP and DCP
Cakmak et al. (2020) [89]	Spectral Domain	Macula: 6 × 6 Disk: 4.5 × 4.5	25/37	↓ vessel density in SCP and DCP; ↑ FAZ of SCP and DCP = density of radial peripapillary capillaries
Dogan et al. (2021) [93]	Spectral Domain	6 × 6	38/40	↓ vascular density in DCP, = in SCP and choriocapillaris
Bacherini et al. (2021) [87]	Spectral Domain	3 × 3	13/13	↓ vascular density in SCP and DCP; = vessel perfusion and FAZ

OCTA = Optical Coherence Tomography Angiography, FD = Fabry Disease, DCP = Deep Capillary Plexus, SCP = Superficial Capillary Plexus, FAZ = Foveal Avascular Zone; ↓ = reduction; ↑ = increase.

## Data Availability

The data that support the findings of this study are available from the corresponding author, MMC, upon reasonable request.
